# The promises of large language models for protein design and modeling

**DOI:** 10.3389/fbinf.2023.1304099

**Published:** 2023-11-23

**Authors:** Giorgio Valentini, Dario Malchiodi, Jessica Gliozzo, Marco Mesiti, Mauricio Soto-Gomez, Alberto Cabri, Justin Reese, Elena Casiraghi, Peter N. Robinson

**Affiliations:** ^1^ AnacletoLab, Dipartimento di Informatica, Università degli Studi di Milano, Milan, Italy; ^2^ ELLIS, European Laboratory for Learning and Intelligent Systems, Milan, Italy; ^3^ European Commission, Joint Research Centre (JRC), Ispra, Italy; ^4^ Environmental Genomics and Systems Biology Division, Lawrence Berkeley National Laboratory, Berkeley, CA, United States; ^5^ Jackson Lab for Genomic Medicine, Farmington, CT, United States

**Keywords:** large language models, protein modeling, protein design, protein engineering, transformers, deep learning

## Abstract

The recent breakthroughs of Large Language Models (LLMs) in the context of natural language processing have opened the way to significant advances in protein research. Indeed, the relationships between human natural language and the “language of proteins” invite the application and adaptation of LLMs to protein modelling and design. Considering the impressive results of GPT-4 and other recently developed LLMs in processing, generating and translating human languages, we anticipate analogous results with the language of proteins. Indeed, protein language models have been already trained to accurately predict protein properties, generate novel functionally characterized proteins, achieving state-of-the-art results. In this paper we discuss the promises and the open challenges raised by this novel and exciting research area, and we propose our perspective on how LLMs will affect protein modeling and design.

## 1 Introduction

Machine Learning (ML) methods have a long-standing history in natural language processing (NLP), and considering the similarities between natural and protein languages (Ofer et al., 2021), NLP methods have been transferred and adapted in the context of protein design and modeling. Indeed, as far back as the 1990s, “shallow” ML methods such as hidden Markov models and support vector machines were applied both in NLP and computational biology (Krogh et al., 1994; Zhou and Su, 2002). Then the application of shallow neural networks for word representation learning (Mikolov et al., 2013) and, more importantly, the advent of deep learning methods introduced significant advances in NLP and in protein modeling (Collobert and Weston, 2008; Manning, 2015; Hou et al., 2017). In particular recurrent neural networks (RNN) displayed excellent performance because of their ability to learn long-range relationships between words as well as between amino acids, and demonstrated to be essential for both global text comprehension and to detect long-range distal contacts in proteins (Socher et al., 2011; Krause et al., 2017).

Recently two main breakthroughs in NLP research led to the so called “foundation models” (Bommasani et al., 2021), a.k.a. Large Language AI Models (LLMs) trained on very large corpora of data through “self-supervised-learning”, i.e., using no or only a small amount of task-specific labelled data.

The first breakthrough is represented by the “attention mechanism” proposed in the Bengio’s seminal paper (Bahdanau et al., 2015) by which the neural machine learns in the translation process the main semantic relationships between words and at a higher level between sentences and paragraphs, by focusing for each word on its most semantically correlated words to improve text comprehension. The second breakthrough is represented by the introduction of the transformer model by Google Brain (Vaswani et al., 2017), that allows a parallel implementation of the Self-Attention mechanism, thus fully exploiting GPU and TPU architectures. Additionally, it can detect relationships between the different words and sentences at any position, without the need of the sequential computation which is inherent to the nature of RNNs. These models, by leveraging their general knowledge acquired from big data, are adaptable to a wide range of downstream tasks, and profoundly differ from conventional learning machines which are usually able to perform only specific tasks for which they have been explicitly trained (Bommasani et al., 2021).

LLMs are expected to revolutionize molecular biology and medicine (Moor et al., 2023). In particular, the relationships between the “language of proteins” and the human natural language motivate the adaptation and application of LLMs, initially conceived for NLP, to relevant protein modeling tasks, such as secondary and tertiary structure prediction, remote homology detection (Rives et al., 2021; Brandes et al., 2022), *de novo* generation of functionally characterized proteins (Madani et al., 2023), design of antibodies that bind to specific ligands (Hie et al., 2023), prediction of protein mutational effects (Ferruz and Hocker, 2022), improvement of the state of the art of proteomics (Elnaggar et al., 2022; Unsal et al., 2022; Olenyi et al., 2023), with relevant applications in medicine, pharmacology, and environmental health (Ferruz and Höcker, 2022).

The next section summarizes the main similarities and differences between human natural languages and protein languages. We then introduce the main structural characteristics of LLMs designed for NLP, and discuss their extension to protein processing and generation. Finally we discuss the exciting perspectives and open problems raised by this promising AI research area in the field of protein modeling and design.

## 2 Natural language and the language of proteins

Analogous to natural language, we can interpret the primary sequence of proteins as a language with its own syntactical rules and semantics (Ofer et al., 2021), wherein the 20 common amino acids plus other unconventional and rare amino acids constitute the letters of the alphabet. Moreover, like natural languages, proteins can be composed of reusable modular elements presenting slight variations that can be rearranged and assembled in a hierarchical structure. Motifs and domains can be related to words and syntactic structures of natural language, while an entire amino acid sequence is analogous to a sentence of a natural language encoding its structure and function. Moreover, multiple polypeptide chains that assemble in a quaternary structure are analogous to sentences that form a longer text. As outlined in Ferruz and Höcker (2022), natural language and proteins have parallel origins and evolution. New words are continuously introduced in languages for expressing new concepts under the pressure of socio-cultural evolution, and natural evolution shapes novel proteins that better fit the environment. Moreover, both natural language words and amino acids are affected by context: their meaning depends on their surrounding elements. Sentences in natural language also present long-distance dependencies (e.g., subjects across sentences in long text). These dependencies are also present in proteins where amino acids distant in the primary structure can be connected in their tertiary and quaternary structure. Adding, removing, or changing a single letter in a natural language sentence can change its meaning or render it meaningless, similar to how a single mutation can cause a loss or gain of function in a protein leading to disruptive pathogenic effects. For example, sickle cell anemia is due to a single sequence change in which a single amino acid (the glutamic acid that is usually in the sixth position of the protein chain) is replaced by a valine in the *β*-globin subunit of the hemoglobin protein. Lastly, crafting a grammatically correct but meaningless sentence bears some resemblance to protein structures that lack any discernible function or may even cause disease, as in the case of amyloid fibrils.

Proteins and natural languages also present differences that need to be taken into account in their processing. In human languages, the alphabet contains many symbols (like uniform punctuation and stop words) (Ofer et al., 2021). In contrast, the alphabet of protein language adopts a simpler alphabet of 20 characters. Nevertheless the letters of proteins can be modified to alter their function, e.g., through methylation of lysine residues, phosphorylation, ubiquitination and other post-translational modifications, thus adding complexity to the protein language. The language of proteins can be described by the use of stochastic context-free grammars (Dyrka and Nebel, 2009) for covering any higher-order dependencies such as nested and crossing relationships that are common in proteins. Human languages define words clearly in written texts, but protein “word boundaries” are less evident because we do not always know *a priori* if a certain sequence is related to a function (e.g., it is part of a domain/motif). One possibility is to use the secondary structure for splitting the sentences into words or to exploit sub-word segmentation that does not require any predefined knowledge of words in the protein language. However, the tokenization process would require exploiting the tertiary structure with more intensive calculations. The overall understanding of the protein language is limited, requiring extensive experimental tests to identify its functionalities. Indeed, even if different corpora exist to train protein language models, the correct interpretation of the produced sequences remains a challenge. Protein evolution differs from language evolution, containing irregularities due to randomness and environmental pressure, and with a grammar that unavoidably will contain many irregularities. Finally, we have to remark on the size of the language of proteins that needs to cover millions of species on Earth, which necessitates studying the general properties of proteins rather than studying the proteins of a particular species.

While the dissimilarities between human and protein languages present significant challenges for applying NLP to protein design, the apparent connections between the two fields offer a new perspective in protein research, opening the way to the adaptation of NLP models to protein modeling and design.

## 3 Large language models for natural language processing

In this section we first discuss the main characteristics of the transformer (Vaswani et al., 2017) and then present two other popular models (BERT (Devlin et al., 2019) and GPT (OpenAI, 2023)) that can be considered an evolution of the original transformer.

### 3.1 The transformer

**FIGURE 1:**
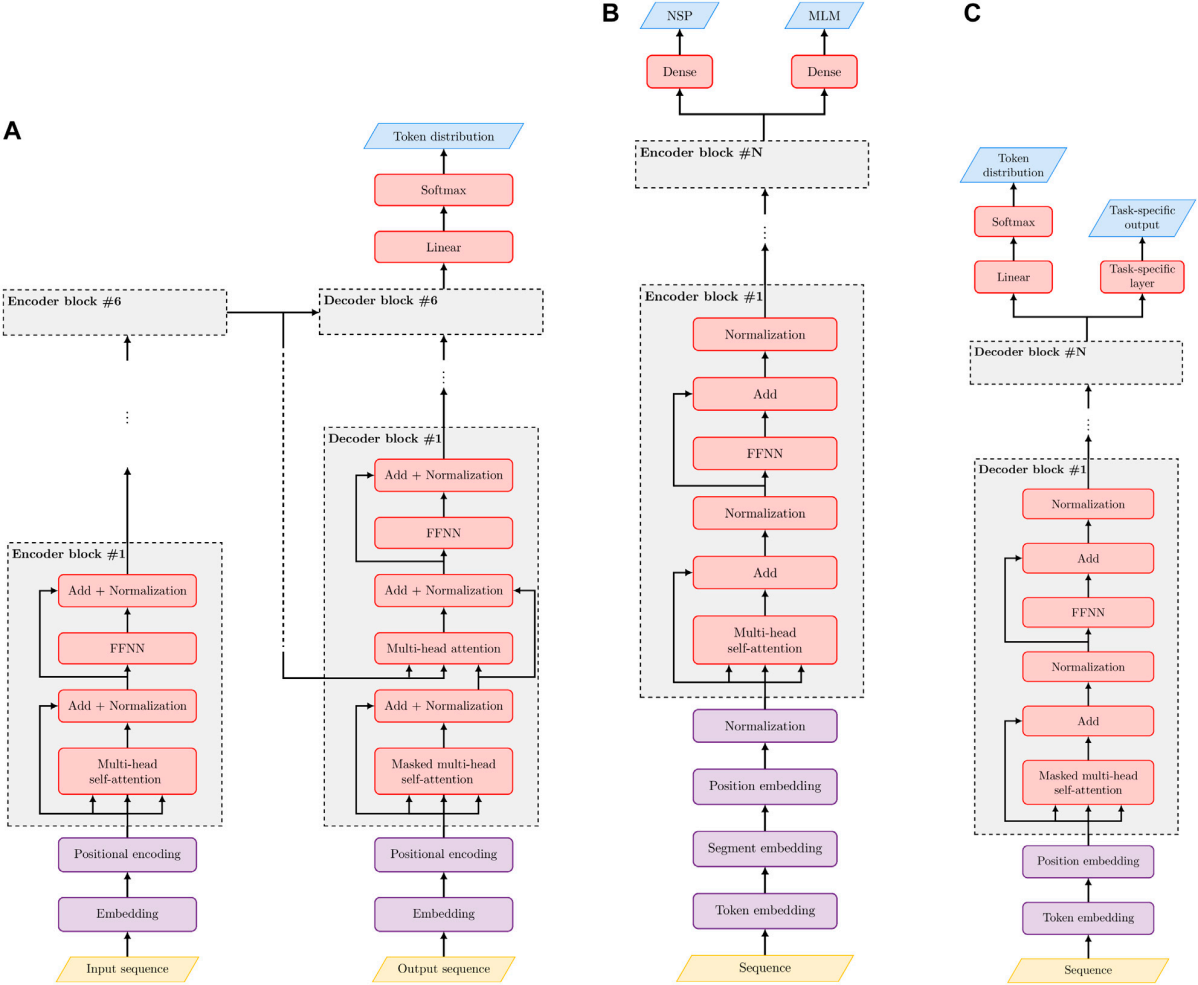
The modular architecture of Transformers. **(A)** The full Encoder-Decoder architecture of the Vaswani et al. (2017) Transformer. **(B)** The Encoder-based BERT Transformer. **(C)** The Decoder-based GPT Transformer. NSP stands for Next-sequence prediction, MLM for Masked Language Model, FFNN for dense Feed Forward Neural Network. Orange parallelograms represent inputs, cyan parallelograms outputs, violet rectangles pre-processing layers and pink rectangles processing layers that implement the submodules of the Encoder and Decoder blocks.

The Transformer is a deep neural network composed of two main components: an Encoder and a Decoder. Both the Encoder and Decoder possess a modular architecture, including a stack of repeated blocks, in which the output of each module is the input of the subsequent one (Figure 1A).

Basically, the Transformer can be applied to translate a text **a** to **t**. However, by changing only the last (top) layers of the network we can construct text classifiers, named entity recognizers, automatic summarizers, and more in general solve a large range of different prediction tasks. Here we introduce the main characteristics of this model. More details are available in the Supplementary Information.

The Transformer is based upon the following main concepts:

• Self-supervised learning: The Transformer learns in a supervised way, but without using explicit labels (Vaswani et al., 2017). This is accomplished by predicting the next element in a sequence, given the previous elements in an autoregressive way (Krishnan et al., 2022). This opens the way to train the model with the large corpus of text data available from the Web (Shwartz-Ziv and LeCun, 2023).
• Multi-task and transfer Learning: The Transformer can learn multiple-tasks at a time (Radford et al., 2019) and can transfer its knowledge, embedded in the model pre-trained with large data sets, to other related learning tasks through fine-tuning or also without using any new task-specific data (zero-shot learning) (Rao et al., 2019; OpenAI, 2023).
• Attention mechanism: This component (Bahdanau et al., 2015) enables the modeling of dependencies between different positions in a text, independently of their distance in the input or output sequence. As we will see more in detail in the next section, through the attention mechanism the embedded representations of each word (i.e., their vectorial representation) in a text include the syntactic and semantic relationships with all the other words in the text itself.
• Self-Attention: While the attention mechanism, originally proposed in the Bengio’s neural translation machine, leverages the relationships between the input words to learn the correspondences between the output words of the translation, the Transformer exploits a similar mechanism to find the semantic relationships between the words of the input sequence, in order to compute a representation of the sequence itself. In this way we can efficiently compute long-range dependencies between the elements of a sequence. See Supplementary Information for more details.
• Multi-head attention: Self-Attention is computed multiple times in parallel using “multiple heads,” in order to capture the different syntactic and semantic relationships among the elements of the sequence.
• Interpretability: A side-effect of Self-Attention is the interpretability of the model. Indeed each attention head can capture different types of syntactic and semantic relationships between the elements of the sequence (Vig, 2019).
• Parallel computation: Instead of processing the elements of a sequence one at a time as in a RNN, Transformers are able to proceed in parallel, thus achieving a substantial speed-up in computation, fully exploiting the parallel computational capabilities of GPUs and TPUs.

Each Encoder block is composed of two stacked sub-modules: 1) the Self-Attention layer and 2) a feed forward neural network (FFNN) with one hidden layer (Figure 1A). Residual connections are used in both sub-modules to counteract the vanishing/exploding gradient phenomenon that plagues deep neural networks (Jastrzebski et al., 2018), and layer normalization across features is finally performed (Ba et al., 2016).

The Decoder basically predicts step by step the translated sentence, receiving as input both the output of the last Encoder layer and the previously predicted words of the Decoder (Figure 1A). During training, all words preceding the one to be predicted are given as input, thus resulting in autoregressive learning. Each Decoder is structured in three layers: 1) a masked multi-head Self-Attention layer; 2) a multi-head attention layer; 3) a FFNN (Figure 1A). The overall structure resembles that of the Encoder, with an additional layer and a masked version of Self-Attention in the first layer. Finally, the Transformer predicts the output sequence step-by-step, since it is able to learn the probability distribution of its tokens, by using a linear and softmax layer on top of the last Decoder block.

### 3.2 BERT and generative pre-trained transformers

Other LLMs have been developed that leverage or extend the Transformer architecture, but the two most successful have likely been Bidirectional Encoder Representations from Transformers (BERT) (Devlin et al., 2019), based on the Encoder component of the Transformer, and the different versions of the Generative Pre-trained Transformer (GPT) (Radford et al., 2018; Brown et al., 2020; OpenAI, 2023) based, instead, on the Decoder component. BERT basically provides a meaningful vector representation of the text, while GPT is mainly a generative model that is able to synthesize novel text. Both models are intensively pre-trained with large text corpora, in order to acquire a general-purpose “linguistic knowledge” that can be successively refined for different specific predictive tasks. This represents a significant difference with respect to previous deep learning models, which are usually focused on specific tasks and are not able to transfer their knowledge in contexts different from those on which they have been specifically trained. For instance, BERT has been pre-trained on about 3.3B words from English Wikipedia and BooksCorpus.

#### 3.2.1 BERT

The architecture of BERT basically consists of stacked Encoder blocks, each containing a Self-Attention and a FFNN layer (Figure 1B). Two types of self-supervised learning tasks characterize BERT pre-training: masked language model (MLM) and next sequence prediction (NSP). In MLM, the input sentence is “masked,” in the sense that 15% of the words are randomly hidden (i.e., they are coded with a <MASK> tag) and predicted at the output of the Encoder. In this way, the model is trained to predict the masked word on the basis of its joint left and right context (in that sense the model is Bidirectional), while the standard Transformer and GPT learn only from the “left” context. This is because BERT basically learns a representation of the text, while GPT, that is essentially a generative model, will predict the next word on the basis of the previous “left” words. At the same time BERT is trained to learn the next sentence (NSP), given the previous one. Indeed, BERT may have in input either one or two sentences (separated by the <SEP> token in the latter case), and the final hidden embedding is used to predict whether the second sentence follows the first one (Figure 1B). For fine tuning several tasks can be learnt starting by putting on top of the pre-trained Encoder a specific learning machine (e.g., a softmax classifier) to train the model to classify sentences or for other tasks, including question answering, summarization, sentiment analysis and many others (Devlin et al., 2019).

#### 3.2.2 GPT

GPT models (Figure 1C) are basically Transformers composed only of stacked Decoder modules, since they are generative models that can learn and predict each element of a sequence on the basis of its previous elements (that is, using only the “left” context—see Supplementary Material for details). Indeed, considering that **x** is a sequence (e.g., a sequence of words in NLP or amino acids in protein modeling), we can factorize the probability *p*(**x**) of observing a sequence of tokens **x** = {*x*_1_, …, *x*_*n*_} using the chain rule, thus decomposing the sequence prediction problem into next-word prediction (Bengio et al., 2003):

$$p\left(x\right)=\overset{n}{\underset{i=1}{\prod }}p\left(x_{i}|x_{<i}\right)\;,$$ (1)

where *x*_<*i*_ denotes the tokens preceding *x*_*i*_. The final softmax layer on top of the last Decoder predicts the probability distribution of the next token of the sequence (Figure 1C), by estimating the parameters *θ* of the deep neural network by stochastic gradient descent to minimize the negative log-likelihood of the factorized probabilities across a training set *X* = {**x** ^1^, **x** ^2^, …, **x** ^|*X*|^} of sequences (Radford et al., 2019):

$$L\left(X\right)=-\overset{\left|X\right|}{\underset{k=1}{\sum }}\overset{\left|x^{k}\right|}{\underset{i=1}{\sum }}\log p_{\theta }\left(x_{i}^{k}|x_{<i}^{k}\right)\;.$$ (2)

Training is performed in two steps: 1) Self-supervised pre-training and 2) Supervised fine-tuning. During self-supervised training the model leverages linguistic information from unlabeled data by learning to predict the next token given the preceding tokens. In the second step, the general-purpose knowledge acquired in the first step is exploited and only a limited set of labeled examples is necessary to fine-tune the model, by adding a task-specific layer to perform prediction in a specialized context (Figure 1C). Using simple task-specific input transformation, without the need to heavily modify the overall architecture of the model, GPT is able to achieve state-of-the-art results on specific tasks ranging from text classification to textual entailment and question answering, and, of course, automatic text generation (Radford et al., 2018).

OpenAI released successive enhancements of GPT, namely, GPT-2 (Radford et al., 2019), GPT-3 (Brown et al., 2020) and recently GPT-4 (OpenAI, 2023), that scaled from 1.5 billion parameters to the huge GPT-4 with likely more than 100 trillion parameters. OpenAI showed that by scaling the original architecture, the Transformer is able to learn and make predictions for new tasks for which have not been specifically trained without a second-level fine-tuning (zero-shot learning) or for which only one or few examples have been provided (one- and few-shot learning). In other words, language modeling with self-supervised learning and Self-Attention using a huge amount of unsupervised text data for training, enables GPT to answer questions, translate texts, and even pass professional and academic exams and perform a large range of learning tasks without an explicit, task-specific training. Moreover GPT-4 can integrate both text and images, thus opening the way to multi-modal self-supervised-learning with LLMs. However, at the current stage (September 2023), despite the revolutionary scenarios opened by these models, there are several limitations and drawbacks as outlined by OpenAI itself and by the scientific community (Bommasani et al., 2021; Mitchell and Krakauer, 2023; OpenAI, 2023).

## 4 Large language models for protein modeling

The success of LLMs for NLP and the similarity between natural language and “protein language” motivated the design of Protein Language Models (PLM), in which, rather than modeling the distribution of words/texts, amino acid and proteins are modeled instead (Rives et al., 2021; Brandes et al., 2022; Ferruz et al., 2022; Ferruz and Hocker, 2022; Madani et al., 2023). Indeed, Transformers can learn interactions between amino acid residues through the Self-Attention mechanism, and by stacking multiple layers they can also learn long-range contexts within sequences in a hierarchical way, thus learning multiple-residue interactions between motifs and domains. Moreover, self-supervised learning is allowed by the availability of large public domain protein repositories, e.g., UniParc and UniProt (Madsen et al., 2022). Table 1 summarizes state-of-the-art main applications of LLMs to protein processing, analysis, modeling and design.

**TABLE 1:** Summary of LLM applications to protein analysis, modeling and design (see text for more details).

Application	Technique	References
Secondary structure and contact prediction, remote homology detection, stability landscape prediction	Pre-trained Encoder-based model and task specific supervised models	Rao et al. (2019)
Prediction of long range conctacts and mutational effects	BERT-based model with deep learning supervised models on top	Rives et al. (2021)
In silico synthesis of antibodies against Ebola and SARS-CoV2 viruses	BERT-based model	Hie et al. (2023)
Secondary structure prediction, remote homology, fold classes and signal peptide predictions, post-translation and biophysical properties prediction	Fine tuned modified BERT model with “local” Encoder for sequence learning and a global “Encoder” for GO annotation learning	Brandes et al. (2022)
Sequence-function protein landscape modeling and generation of high-fitness sequences	Encoder-based tansformer coupled with dimensionality reduction techniques, deep convolutional and dense FFNN	Castro et al. (2022)
Protein secondary structure and sub-cellular location prediction	Auto-regressive and Encoder-based models	Elnaggar et al. (2022)
*De-novo* generation of protein sequences similar to natural ones, and generation of novel proteins unexplored by natural evolution	GPT-2 based model	Ferruz and Schmidt (2022)
Design of novel stable protein structures	Decoder-based generative model	Moffat et al. (2022)
Function loss and mutational effect prediction	Decoder-based transformer and downstream supervised models	Ferruz and Höcker (2022)
In silico generation of functionally characterized proteins	Conditional Transformer	Madani et al. (2023)
Engineering of specific heavy- and light-chain antibodies	Conditional Transformer	Shuai et al. (2022)
Prediction and generation of the binding target of a protein	Full Encoder-Decoder transformer	Grechishnikova (2021)
Generation of enzymes that catalyze the chemical reactions of specific reactants	Full Encoder-Decoder transformer	Schwaller et al. (2021)
Inverse folding prediction	Encoder-Decoder transformer model combining sequence and structural data	Heinzinger et al. (2023)

### 4.1 Encoder-based protein language models

The first proposed PLMs adopted an Encoder-only Transformer architecture, since their aim was to obtain embedded representations of proteins in a vector space for downstream tasks. For instance, TAPE (Task Assessing Protein Embedding) has been pre-trained to obtain embeddings, which have subsequently been processed via different supervised models in order to solve several downstream tasks (secondary structure and contact prediction, remote homology detection, fluorescent landscape and stability landscape prediction) (Rao et al., 2019). Rives et al. (2021) proposed ESM, a BERT-based model trained on 250M protein sequences with 33 layers, able to encode the properties of the proteins at different hierarchical levels, from their evolutionary relationships to the biochemical and biophysical properties of amino acids. Using deep learning models on top of the embedded protein representations, the authors achieved state-of-the-art predictions on long-range contacts and mutational effects. The same model has been applied to efficiently evolve human antibodies by suggesting evolutionarily plausible mutations, resulting in antibodies with improved binding affinity and activity against Ebola and SARS-CoV2 viruses (Hie et al., 2023).

Other models modified the original BERT Encoder-Transformer to better represent the protein world. For instance, ProteinBERT obtains “functionally aware” protein representations by simultaneously learning the protein sequences in the “local” Encoder stacked modules and their GO annotations in the “global” Encoder stacked modules. The local and global modules are trained in parallel and the former representations affect the latter ones through a global Attention module, while global representations influence the local ones through fully connected dense layers. The pre-trained models are then fine-tuned on several downstream tasks, ranging from secondary structure prediction, to remote homology, fold classes and signal peptide predictions, as well as post-translation and biophysical properties prediction, using only a fully connected dense layer on top of the Encoders (Brandes et al., 2022).

Another model that significantly modifies the original BERT Transformer is represented by Regularized Latent Space Optimization (ReLSO) (Castro et al., 2022). Its Encoder blocks are coupled with innovative dimensionality reduction techniques based on the Attention mechanism, deep convolutional and dense FFNN, to effectively model the sequence-function protein landscape and generate high-fitness sequences (Castro et al., 2022).

### 4.2 Decoder-based generative protein language models

Decoders are generative models which learn to predict the next amino acid, given the previous ones in the sequence by using masked Self-Attention layers (Section 3.1; Supplementary Material). In this sense, they are generative since in the prediction stage they are able to output a new amino acid at a time.

One of the most representative methods of these generative approaches is ProGPT2 (Ferruz and Hocker, 2022). The authors showed that this model, based on the GPT-2 architecture with 738M of parameters and trained on about 50M of proteins drawn from Uniref50 clustered sets of UniProtKB sequences, can not only *de novo* generate protein sequences similar to natural ones, but can also explore protein regions unexplored by natural evolution. The new sequences, despite their relative sequence diversity compared to naturally occurring proteins, show structural similarity, predicted stability and several common properties with proteins sampled by natural selection (Ferruz and Hocker, 2022).

Differently from the previous approach, that relies on natural sequences, Design in Areas of Restricted Knowledge (DARK) is a *de novo* Decoder-based protein design method with 110M parameters, trained on synthetic sequences (Moffat et al., 2022). The authors showed that through this approach we can design novel stable and ordered structures (as judged by AlphaFold2 (Jumper et al., 2021)). Note that, despite the fact that both ProGPT2 and DARK are basically generative models, they provide vector representations of proteins in their last layer, and as such these representations can be given as input to downstream models (for instance, deep neural networks) to predict, e.g., function loss or mutational effects (Ferruz and Höcker, 2022).

### 4.3 Conditional transformers for the design of functionally characterized proteins

One of the main objective of protein engineering is the generation of proteins having specific properties or desired functionalities for applications in pharmacology, medicine and environmental health (Li et al., 2020). From this standpoint, conditional Transformers open new perspectives for tailored protein design. Leveraging basically the same LLM originally designed for conditional text generation (Keskar et al., 2019), the Progen model can generate functionally characterized proteins by including functional tags during training (Madani et al., 2023). The Progen generative model is a Decoder composed by 36 stacked layers, with 8 Self-Attention heads for each layer and a total of 1.2G of trainable neural network parameters. It receives in input not only a context sequence of amino acids, but also a functional tag *f* representing, e.g., a GO biological process, a molecular function or a protein family, or whatever property of the protein, thus decomposing the sequence prediction problem into next-amino acid prediction problem, instead of next-word prediction (as in Eq. 1), but this time also conditioned on *f*:

$$p\left(x|f\right)=\overset{n}{\underset{i=1}{\prod }}p\left(x_{i}|x_{<i},f\right)\;.$$ (3)

The objective function to be minimized is analogous to that of Eq. 2, where now **x** ^*k*^ represents a protein:

$$L\left(X\right)=-\overset{\left|X\right|}{\underset{k=1}{\sum }}\overset{\left|x^{k}\right|}{\underset{i=1}{\sum }}\log p_{\theta }\left(x_{i}^{k}|x_{<i}^{k},f^{k}\right)\;,$$ (4)

where now conditioning is done on the functional tag *f* ^*k*^ associated with the protein **x** ^*k*^. The functional tag provides a point of control over the generation process, and it constraints the protein generation toward proteins having a specific property *f* ^*k*^. Indeed, in their work, Madani et al. (2023) showed that Progen can generate novel proteins that show similar functional and structural characteristics of natural proteins on the basis of the provided functional tags.

On the same research line, an Immunoglobulin Language Model (IgLM) has been developed by training on about half a billion of antibody heavy- and light-chain variable sequences, and conditioning on species of origin and chain type, thus opening the way to PLM-based engineering of specific antibodies (Shuai et al., 2022).

### 4.4 Encoder-decoder transformers for *de novo* drug generation

One of the natural and most successful applications of full Encoder-Decoder Transformers in NLP is language translation (Tan et al., 2020). Following the same principle of transforming a text into another corresponding text, we can “translate” a protein into its ligand, or, *vice versa*, given a ligand we could generate its corresponding protein binder. This is the approach proposed by Grechishnikova (2021), that applied the original Encoder-Decoder Transformer architecture (Vaswani et al., 2017) to translate a protein into its corresponding ligand in SMILE format (Weininger, 1988): the Encoder computes a protein embedding, while the Decoder generates the corresponding binding target. By reversing the inputs and outputs of the Transformer we could in principle obtain the retrosynthesis of a protein for a given ligand given as input to the translation machine, following a general approach proposed by IBM researchers to generate the reactants needed to synthesize a molecule given as input to the Transformer (Schwaller et al., 2020). This approach opens the way to the *in silico de novo* engineering of protein drugs that bind to specific molecular targets. Moreover, by extending and adapting to the protein world recent IBM research on Attention-based neural networks for mapping the chemical space (Schwaller et al., 2021), we could design Encoder-Decoder Transformers able to generate enzymes (output of the Decoder) that catalyze the chemical reactions of specific reactants (input of the Encoder), with possible applications in pharmacology, or in environmental health.

## 5 Discussion

LLMs learn the probability distribution of the elements in a sequence (e.g., amino acids inside proteins) and are able to do this by using self-supervised learning, i.e., by exploiting the pure unannotated protein sequences massively available in public repositories. From this standpoint, they are “general-purpose learners” in the sense that having learnt the protein distribution (if sufficient data are available and the model is sufficiently large), they can make predictions on tasks for which they have not been specifically trained or can be secondarily trained on specific tasks using only limited supervised fine tuning [as in the Lysozyme protein family prediction with ProGen (Madani et al., 2023)]. Such foundation models (Bommasani et al., 2021), with enhanced modeling capabilities, are thus expected to solve a large range of complex problems in medicine and molecular biology (Moor et al., 2023), by exploiting their “connectionist knowledge,” embedded in the parameters of the deep neural model.

**FIGURE 2:**
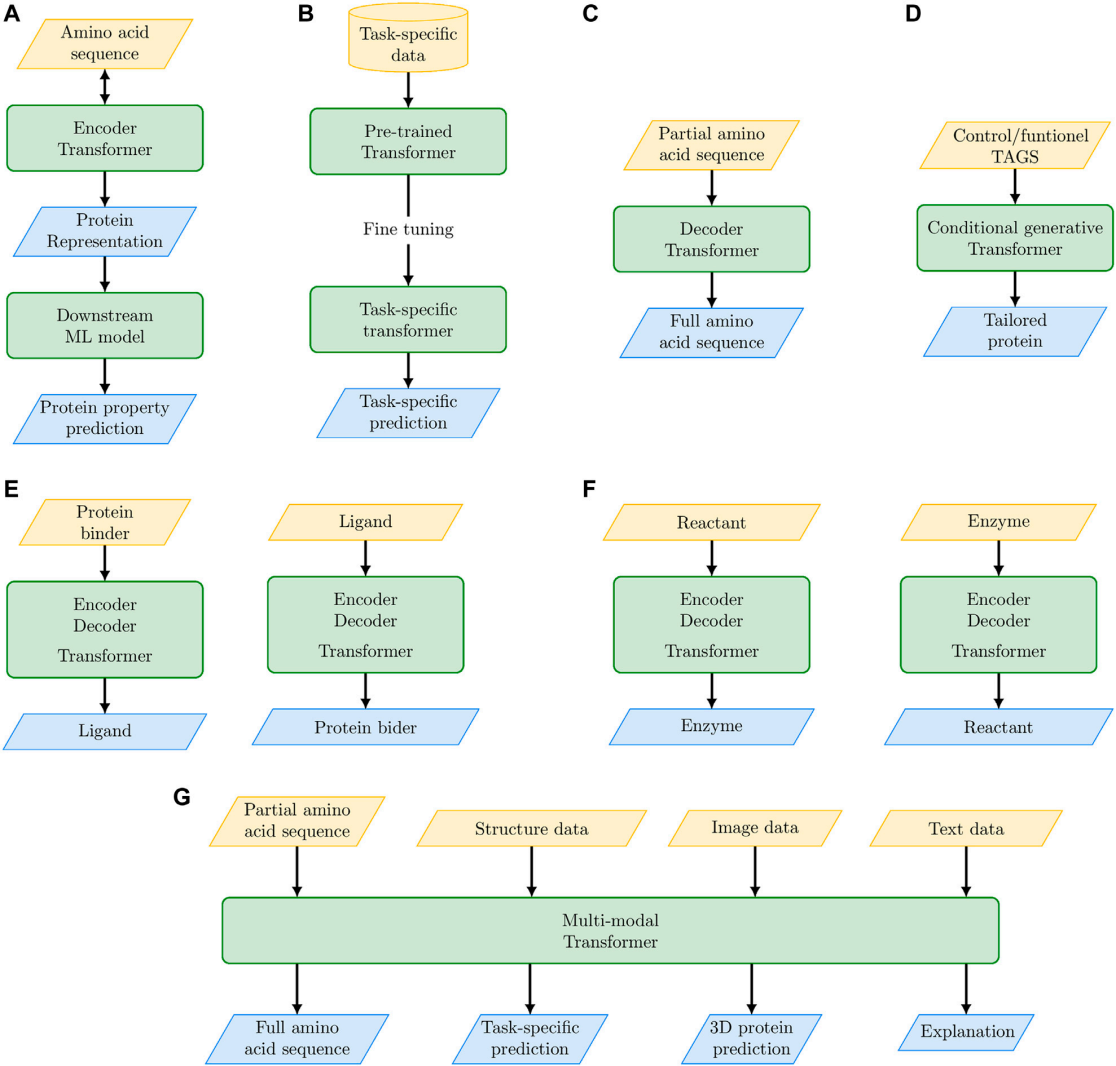
The “promises” of large language models: their main achievements and their future perspective outcomes for protein modeling and design. **(A)** Protein embedding with Encoder-based Transformer coupled with a second level ML model for downstream specific tasks. **(B)** Fine tuning of pre-trained Transformer on specific protein tasks. **(C)** Unconditioned protein generation with Decoder-based Transformers. **(D)** Conditional generative Transformers for tailored protein design. **(E)** Encoder-Decoder Transformer for *de novo* drug design. **(F)** Encoder-Decoder Transformer for the design of enzymes that catalyze specific biochemical reactions. **(G)** Multi-modal Transformers to integrate multiple sources of data for solving complex protein modeling problems.

The main achievements and possible future outcomes of PLMs are schematically summarized in Figure 2; Table 2. The embedded protein representations generated by Encoder-based Transformers represent the input for supervised or unsupervised ML models for downstream tasks (e.g., protein classification, mutational effect prediction, Figure 2A). Transformers pre-trained on a large corpus of proteins can be specialized to model a specific set of proteins (e.g., the family of translation initiation factors) by fine-tuning on that specific set (Figure 2B). Decoder-based Transformers can generate novel proteins in an unconditioned way (Figure 2C) or functionally characterized proteins by using control tags (Figure 2D). The full Encoder-Decoder Transformer architecture can be used to predict ligands of possible protein binders (or *vice versa*, Figure 2E), or it can be used to design enzymes for specific biochemical reactions (Figure 2F). In perspective, we can envision multi-modal PLMs that by integrating multiple sources of data (not exclusively sequence data) can not only solve complex protein modeling problems, but also explain the reasons underlying their predictions (Figure 2G).

**TABLE 2:** Summary of prospective applications of LLMs.

Application	Technique
General-purpose learning of sequence, structure, features and functional characteristics of proteins	Foundation models trained on huge corpora of protein data
Breakthrough enhancements of classical prediction problems in proteomics (e.g., protein and isoform function classification, mutational effect prediction)	Encoder-based transformers coupled with downstream specialized supervised learners
Generation of novel proteins functionally characterized that enlarge the landscape of their natural evolution	Pre-trained conditional transformers fine-tuned on specific functionally characterized set of proteins
Prediction and automatic generation of a protein target and prediction of a protein given a specific target	Full Encoder-Decoder transformer or Generative Decoder models
Design of enzymes for specific biochemical reactions	Encoder-Decoder transformer
*De-novo* drug design	Full Encoder-Decoder transformers constrained with structural data
Solving complex modeling problems in proteomics and drug design	Integrative multi-modal transformers combining sequence, imaging, text and structural data
Explainable and interpretable PLMs	Post-hoc methods; attention-based visual explanation transformer models; GPT “interpreting” PLMs
Reduction of the complexity of PLMs with limited performance decay	Neural network compression techniques: e.g., pruning, quantization, distillation; compression-oriented modified transformer models

Indeed, an open problem posed by PLMs and more in general by LLMs is their explanation and interpretability. Given the increasing and widespread usage of LLM to solve problems involving high stakes decisions, we need to generate both global explanations, to provide hints about the generalized rules inferred by the model and its behavior as a whole, and local explanations, to interpret specific predictions (Rudin, 2019).

To this aim, *post hoc* methods (Madsen et al., 2022), which work on the already trained model and are often model-agnostic, could be in principle applied to explain PLMs. Among them, in the context of text classification classic perturbation-based local-explanation methods (the most famous being LIME (Ribeiro et al., 2016), Anchors (Ribeiro et al., 2018), SHAP (Lundberg and Lee, 2017)) have been already combined (Szczepański et al., 2021), or modified (Kokalj et al., 2021) to better deal with Transformers models, to provide scores assessing the impact of each token on the predicted class-probability.

A new trend of research, specifically focused on Transformer explainability, is instead evaluating the crucial influence of Attention in the produced output sequence and is therefore focusing on providing interactive Attention-based visual-explanations. Examples of these attempts are exBert (Hoover et al., 2020) and BertViz (Vig, 2019). Though the *faithfulness* and *plausibility* (Jacovi and Goldberg, 2020) of explanations provided by computed attentions is an open issue (Bibal et al., 2022), we believe exBert and BertViz are first, promising attempts to lay the foundation for a new set of interactive visualization approaches that might in future provide important hints about the “reasoning” of complex LLMs.

A completely different, and somehow surprising, interpretation approach uses a GPT model to interpret the functions of neurons (based on their activations) in another GPT model (Bills et al., 2023). Though the authors themselves outline the limitations of their work, we believe their proposal is a new promising way to not only interpret the output of complex LLMs, but to also answer the open debate about “whether and how” LLMs are performing some inductive/deductive reasoning based on their connectionist and importance based learning structure (Bender et al., 2021). Other promising approaches in the area of explainability of LLMs in protein function prediction include (Wenzel et al., 2023; Zhou et al., 2023). In particular in (Wenzel et al., 2023) the authors extended the XAI method of Integrative Gradients to inspect the latent amino acid representations in Transformer models in order to discover the relevance of each amino acid for protein function prediction. Moreover the authors showed that the relevant sequence regions were correlated with known functional regional annotations in sequence databases.

Another open issue is represented by the complexity of PLMs, that often requires costly special purpose hardware resources to train, or even query, a LLM/PLM. A possible solution could be the adoption of neural network compression techniques, such as pruning, quantization or distillation, to obtain thinner models once a LLM has been trained. Some experiments show the viability of these approaches, that in some case can attain a reduction of two orders of magnitude for the model size, at the price of a 1% drop in accuracy (Ganesh et al., 2021). However, these techniques have been applied only to specific Transformer-based architetures [see, e.g., Sanh et al. (2019)], and in any case they require as a starting point a LLM induced via standard (i.e., costly) techniques. A very promising, though inexplored, solution might reside in modifying the learning algorithm of Transformer-based models, so that it outputs models that are directly akin to compression (Carreira-Perpiñán and Idelbayev, 2021), or the design of generative models constrained by the 3D structure of the protein (Rao et al., 2021).

In perspective, PLMs could generate synthetic libraries of functionally characterized proteins that can be used to discover, e.g., novel enzymes for industrial applications, or novel candidate drugs conditioned on specific functional characteristics. In particular, conditional Transformers, by using multiple functional tags, can expand the space of protein sequences beyond those sampled by the natural evolution. For instance we can condition protein generation on a functional tag for a specific enzymatic reaction and at the same time on another tag for a specific binding domain, thus generating proteins able to drive a specific biochemical reaction in a specific micro-environment. The capability of processing multi-modal data, i.e., not only sequence or functional tags, but also three-dimensional structures, images and bio-medical text could lead to multi-modal PLMs for precise *de novo* design of proteins, and more in general to solve complex problems in pharmacology, medicine, and environmental health. At this stage (September 2023), these possible outcomes mostly represent an attractive promise, but it is also true that in many fields, including biomolecular biology and medicine, the development and results of novel AI models exceeded any previous forecasting (Jumper et al., 2021; Moor et al., 2023).

## Data Availability

The original contributions presented in the study are included in the article/Supplementary Material, further inquiries can be directed to the corresponding author.
