# Supplementary material for: The promises of large language models for protein design and modeling
Source: Front Bioinform. 2023 Nov 23;3:1304099. doi: 10.3389/fbinf.2023.1304099 (PMC10701588; doi:10.3389/fbinf.2023.1304099)
Supplement: Supplementary file 1 [file DataSheet1.PDF]

The promises of Large Language Models for protein design and modeling - Supplementary Information

Giorgio Valentini[1,2*], **Dario Malchiodi**[1], **Jessica Gliozzo**[1], **Marco Mesiti**[1], **Mauricio Soto-Gomez**[1], **Alberto Cabri**[1], **Justin Reese**[3], **Elena Casiraghi**[1,2,3], **Peter Robinson**[4]

[1] *AnacletoLab, Dipartimento di Informatica, Università degli Studi di Milano, Italy*
[2] *ELLIS, European Laboratory for Learning and Intelligent Systems*
[3] *Environmental Genomics and Systems Biology Division, Lawrence Berkeley National Laboratory, Berkeley, USA*
[4] *Jackson Lab for Genomic Medicine, Farmington, USA*

Correspondence*:
Corresponding Author
valentini@di.unimi.it

S1 STRUCTURE AND IMPLEMENTATION OF THE TRANSFORMER

The Transformer (Vaswani et al., 2017) is composed by two main parts: an Encoder and a Decoder, both showing modular architecture, with a stack of repeated blocks, in which the output of each block is the input of the successive one (Fig. S1) [1]. The initial input of the learning machine is a sequence of symbols $\mathbf{a} = [a_1, a_2, \ldots, a_n]$, where a_i may represent, e.g., a word in NLP problems or an amino acid in protein modeling problems, or a music event in symbolic music processing tasks. Although Transformers are machines able to learn any sequence, historically they have been designed and applied to NLP, and for this brief overview of the model we refer to NLP problems and tasks. The final output of the Transformer is a sequence of symbols $\mathbf{t} = [t_1, t_2, \ldots, t_m]$ representing, e.g., some text or a protein.

Basically, the Transformer can be applied to translate a text $\mathbf{a}$ to $\mathbf{t}$, but changing only the last (top) layers of the network we can construct, e.g., a classifier, or we can solve other prediction tasks, by leveraging the general knowledge and reasoning capabilities embedded in the machine.

S1.1 Encoder.

An encoder block is composed by two stacked submodules: a) the Self-Attention layer and b) a feed forward neural network (FFNN) with one hidden layer (Fig. S1). Residual connections are used in both submodules to counteract the vanishing/exploding gradient phenomenon that plagues deep neural networks (Jastrzebski et al., 2018), and layer normalization across features is finally performed (Ba et al., 2016).

At first we need to tokenize text and represent words as a vectors of real numbers to input them to the encoder through an embedding function $e : A \rightarrow E$, where A is the set of words/elements of the sequence, $E \subset \mathbb{R}^d$ and d is the dimension of the embedded word. The authors here used Byte pair

[1] To make the text more readable, we report here the same scheme of the Transformer used in the main paper

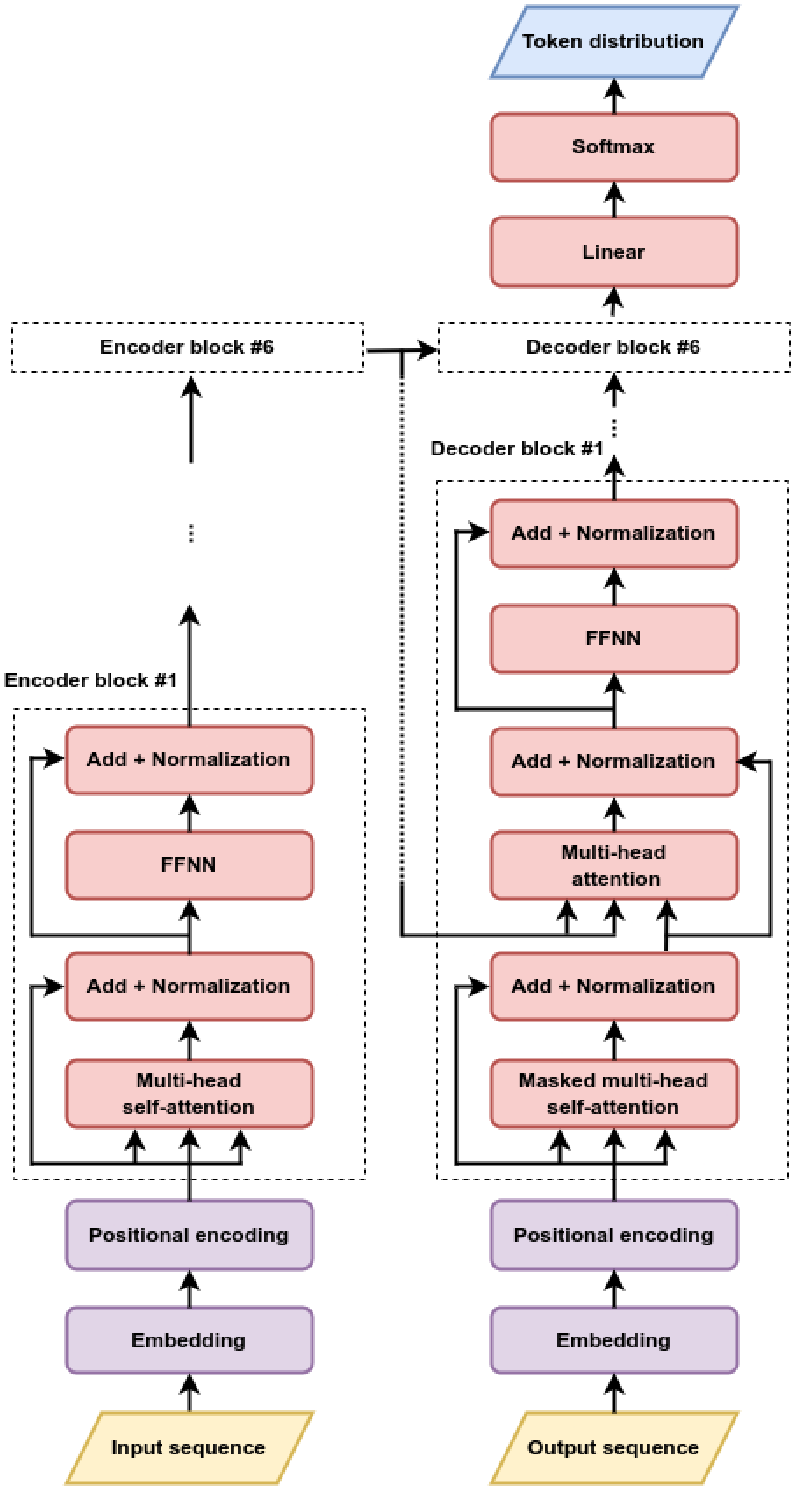

Figure S1. The modular architecture of the full Encoder-Decoder architecture of the Transformer described in Vaswani et al. (2017). Orange parallelograms represent inputs, cyan parallelograms outputs, violet rectangles pre-processing layers and pink rectangles processing layers that implement the submodules of the encoder and decoder blocks.

embedding (Sennrich et al., 2016). A further preprocessing is done by adding a specific positional encoding to all word embeddings, in order to add to the Transformer input also the relevant information contained in the position that each word has in the input sequence (Chen et al., 2021). As a result, the encoder input is a

matrix $X \in \mathbb{R}^{n \times d}$, where n is the maximum number of words in the text and d the dimension of the word encoding. Texts smaller than n words can be processed using padding. The encoder output generates a set of word embeddings $Z \in \mathbb{R}^{n \times d}$ of the same dimension of X.

S1.1.1 The Self-Attention layer.

This layer processes the input $X \in \mathbb{R}^{n \times d}$ by three linear transformations represented by matrices W_Q, W_K and W_V, whose weights are computed as part of the backpropagation algorithm:

$$Q = XW_Q \quad \text{with} \quad Q \in \mathbb{R}^{n \times d'} \quad \text{and} \quad W_Q \in \mathbb{R}^{d \times d'} \quad , \tag{1}$$

$$K = XW_K \quad \text{with} \quad K \in \mathbb{R}^{n \times d'} \quad \text{and} \quad W_K \in \mathbb{R}^{d \times d'} \quad , \tag{2}$$

$$V = XW_V \quad \text{with} \quad V \in \mathbb{R}^{n \times d'} \quad \text{and} \quad W_V \in \mathbb{R}^{d \times d'} \quad . \tag{3}$$

The resulting query matrix Q is multiplied by the key matrix K:

$$A = QK^T \qquad \text{with} \qquad A \in \mathbb{R}^{n \times n} \quad . \tag{4}$$

Each entry of the matrix A represents the syntactic/semantic contribution of each word with respect to another word in the text. This can be better understood by considering the embedding of a specific single word. Indeed, each row of the matrix Q represents a d' dimensional embedding of a word. Note that d is the dimension of the input vector, while d' is the dimension of the vector obtained by the linear transformations of eq. 1, 2 and 3. This embedded vector is multiplied by the key embedding of each other word (eq. 4). If $\mathbf{q}_i$ and $\mathbf{k}_i$ represent the embedding of the ith word, aka the ith row of the matrices Q and K, then we have:

$$\mathbf{a}_i = [a_{i1}, a_{i2}, \ldots, a_{in}] = [\mathbf{q}_i \cdot \mathbf{k}_1, \mathbf{q}_i \cdot \mathbf{k}_2, \ldots, \mathbf{q}_i \cdot \mathbf{k}_n] \quad . \tag{5}$$

Recalling that the dot product represents a similarity between vectors, the ith row $\mathbf{a}_i$ of matrix A represents how much the other words in the text "correspond" to the word in position i. In other words, we can evaluate how much the query $\mathbf{q}_i$ is "aligned" with the keys $\mathbf{k}_j$ that represent the other words in the text. Then a softmax function σ is applied to the elements of matrix A to resemble a probability distribution:

$$\sigma(a_{ij}) = \frac{e^{a_{ij}}}{\sum_{k=1}^{n} e^{a_{ik}}} \quad . \tag{6}$$

Finally, the value matrix V, whose rows $\mathbf{v}_j$ represent how the value embeddings of the different words are weighted through eq. 6, thus resulting in a new embedded representation $\mathbf{z}_i$ of ith word:

$$\mathbf{z}_i = \sum_{j=1}^{n} \mathbf{v}_j \sigma(a_{ij}) = \sum_{j=1}^{n} \mathbf{v}_j \sigma(\mathbf{q}_i \cdot \mathbf{k}_j) \quad . \tag{7}$$

The key point here is that the embedding $\mathbf{z}_i$ of the ith word depends on the embeddings $\mathbf{v}_j$ of all the other words, weighted through the relationships of the ith word with all the words in the text. In other words, the representations of each word "pay attention" to the other words in the text, focusing on those that are more significant, independently of its distance to all the other words in the text itself. This can be compactly expressed in matrix form:

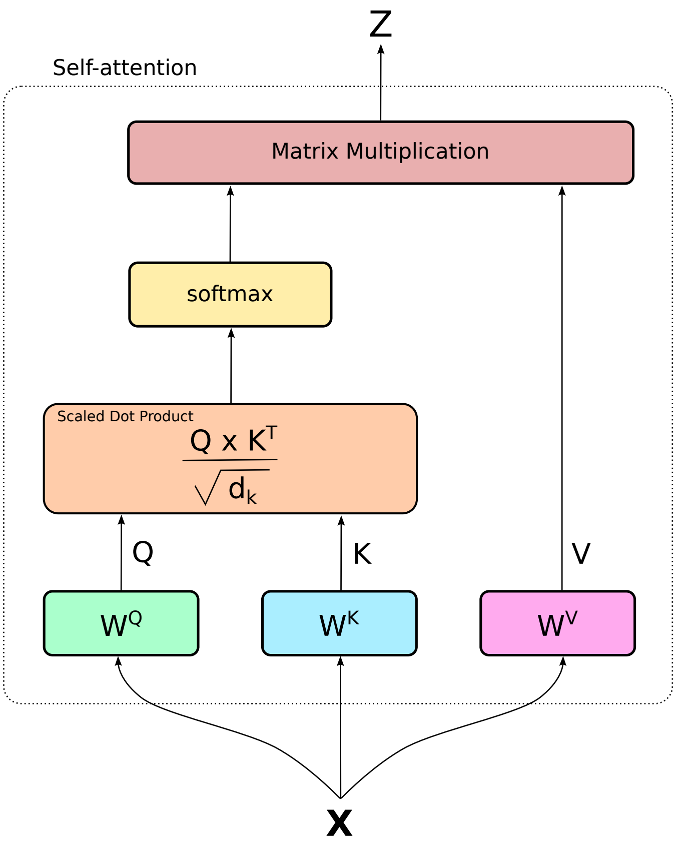

Figure S2. The Self-Attention mechanism of the Transformer.

$$Z = \sigma\left(\frac{QK^T}{\sqrt{d'}}\right)V \quad . \tag{8}$$

The division by $\sqrt{d'}$, where d' is the dimension of the embedding, makes the computation of the gradient more stable. The matrix $Z \in \mathbb{R}^{n \times d'}$ is the output of one "head" of the Self-Attention layer (Fig. S2). In the Transformer model we may have h of these heads (in the original paper $h = 8$), working in parallel, thus implementing the so called "multi-head attention". More precisely, at the end we have h different output matrices $Z_1, \ldots, Z_h$, that will be concatenated and multiplied by a weight matrix W_0:

$$\bar{Z} = [\mathrm{Concat}(Z_1, Z_2, \ldots, Z_h)]\, W_0 \tag{9}$$

Note that if we choose (as in the original paper) $d = hd'$, having $W_0 \in \mathbb{R}^{d \times d}$, we will have $\bar{Z} \in \mathbb{R}^{n \times d}$. Hence, the final embedding is the result of the concatenation of the h Self-Attention heads multiplied by W_0, whose weights are learnt by back propagation.

Summarizing, the Self-Attention mechanism is implemented through a set of matrix multiplications that are able to capture the relationships between the different elements of the sequence.

S1.1.2 Position-wise Feed Forward Neural Network.

The output $\bar{Z}$ of the Self-Attention layer is the input for a FFNN with one hidden layer. Note that the FFNN is fed in parallel with the rows $\bar{\mathbf{z}}_i$ of $\bar{Z}$:

$$\underset{i=1,\dots,n}{\text{Parallel}}\left\{\text{FFNN}(\bar{\mathbf{z}}_i) \rightarrow \tilde{\mathbf{z}}_i\right\} \ . \tag{10}$$

The weights are shared across the n FFNNs, obtaining the final embedding representation $\tilde{Z} \in \mathbb{R}^{n \times d}$.

It is worth noting that multiple encoders are stacked ($N = 6$ stacked encoders are used in Vaswani et al. (2017)) to allow a detection of the relationships between the elements of the sequence at different levels of granularity. At each step the computed $\tilde{Z}$ includes a higher level representation of the relationships between the elements (words) of the text. In analogy with the other layers of the encoder, the FFNN output is post-processed via a residual connection and undergoes layer normalization, thus obtaining the final normalized output $\tilde{Z}' \in \mathbb{R}^{n \times d}$:

$$\tilde{Z}' = \text{Norm}(\tilde{Z} + \bar{Z}) \ . \tag{11}$$

S1.2 Decoder

The Decoder basically predicts step by step the translated sentence, receiving as input both the output of the last Encoder layer and the previously predicted words of the Decoder (Fig. S1). During training all the words preceding the next word to be predicted are given as input thus resulting in an autoregressive learning.

A decoder block is structured in 3 submodules: a) a masked multi-head Self-Attention layer; b) a multi-head attention layer; c) a FFNN (Fig. S1). The overall structure resembles that of the Encoder, with an additional layer, and using as first layer a masked Self-Attention version. The words given in input to the decoder undergo the same preprocessing of Sect. S1.1, namely word embedding and positional encoding.

S1.2.1 Masked multi-head Self-Attention layer.

This layer performs the same Self-Attention mechanism performed by the Encoder, but this time all the elements successive to each word are not considered by masking them. In this way, the neural network cannot see the subsequent words, thus avoiding that the machine trivially copies the next element of the sequence during training. This is accomplished by adding to eq. 8 a matrix $M \in \mathbb{R}^{n \times n}$ whose upper triangular elements are set to $-\infty$:

$$Z = \sigma\left(\frac{QK^T}{\sqrt{d'}} + M\right) V \ . \tag{12}$$

In this way, the upper triangular elements of the matrix $\sigma(QK^T/\sqrt{d'} + M)$ are set to 0, since $e^{-\infty} = 0$, and as a consequence the words of the elements successive to the current one are ignored (i.e., they are masked).

S1.2.2 Multi-head attention layer.

In this layer, a pure attention mechanism is performed, that is we consider the relationships between the input sequence processed by the Encoder to translate the next word in the Decoder block. Indeed, the input of this layer is the output $\tilde{Z}$ of the Encoder, from which the key and value embedding matrices

of the decoder K^D and V^D are obtained, while the query matrix Q^D comes from the Decoder masked Self-Attention layer:

$$K^D = \tilde{Z} W_K^D \qquad \text{and} \qquad V^D = \tilde{Z} W_V^D \ . \tag{13}$$

The decoder learns from the masked input of the previous decoded layer, but it also learns from the full relationships between all the elements of the sequence processed by the Encoder. Note that in this case $Z^D = \sigma\left(Q^D K^{D^T}/\sqrt{d'}\right) V^D$ may have dimension $m \times d$, with $m \neq n$. In other words, the maximum length m of the output sequence can differ from the maximum length n of the input sequence, since $Q^D \in \mathbb{R}^{m \times d'}$.

After passing through the FFNN, we obtain a matrix $\tilde{Z}^D$, similar to that of the $\tilde{Z}$ of the Encoder, but with a number of rows m that can differ from the number of rows n of $\tilde{Z}$.

S1.2.3 Final output layer.

Analogously to the Encoder, also Decoder blocks can be stacked, and on top of the last one a linear layer with softmax computes the probability distribution P of the output words/elements:

$$P = \sigma(\tilde{Z}^D W_P) \tag{14}$$

with $\tilde{Z}^D \in \mathbb{R}^{m \times d}$, $W_P \in \mathbb{R}^{d \times v}$, and $P \in \mathbb{R}^{m \times v}$, where v is the dimension of the output vocabulary. Eq. 14 expresses the predicted probability distribution computed by the Transformer for each element/word of the vocabulary. More precisely, the output of the Transformer is the estimated probability distribution of the words in the vocabulary for each position of the output text.

The loss function that is minimized by the backpropagation algorithm is the cross-entropy or the Kullback-Leibler divergence between the "true" probability distribution and the probability P estimated by the Transformer.

At prediction time we can decode the output using a greedy approach, i.e., choosing each time the word w_i predicted at position i with the maximum probability:

$$w_i = \arg\max_j p_{ij} \tag{15}$$

where p_{ij} are the elements of the estimated probability matrix P. Other algorithms can be used instead: e.g. in the original paper the authors used beam search, that basically tries multiple solutions starting from each of the top k top ranked words and choosing the one that results in the best joint probability (Freitag and Al-Onaizan, 2017).

It is worth noting that the Transformer, being able to learn the probability distribution of the elements of a sequence and usually being trained on a large corpus of data, can be applied to large set of prediction problems in NLP, ranging from text classification, to name entity recognition, question answer, summarization, translation and language modeling.

REFERENCES

Ba, J., Ryan, J., and Hinton, G. (2016). Layer normalization. *ArXiv* abs/1607.06450

Chen, P.-C., Tsai, H., Bhojanapalli, S., Chung, H.-W., Chang, Y.-W., and Ferng, C.-S. (2021). A Simple and Effective Positional Encoding for Transformers. In *Proceedings of the 2021 Conference on Empirical*

Methods in Natural Language Processing (Online and Punta Cana, Dominican Republic: Association for Computational Linguistics), 2974–2988. doi:10.18653/v1/2021.emnlp-main.236

Freitag, M. and Al-Onaizan, Y. (2017). Beam search strategies for neural machine translation. In *First Workshop on Neural Machine Translation, Association for Computational Linguistic*

Jastrzebski, S., Arpit, D., Ballas, N., Verma, V., Che, T., and Bengio, Y. (2018). Residual connections encourage iterative inference. In *International Conference on Learning Representations*. 1–14

Sennrich, R., Haddow, B., and Birch, A. (2016). Neural machine translation of rare words with subword units. In *Proceedings of the 54th Annual Meeting of the Association for Computational Linguistics (Volume 1: Long Papers)* (Berlin, Germany: Association for Computational Linguistics), 1715–1725. doi:10.18653/v1/P16-1162

Vaswani, A., Shazeer, N., Parmar, N., Uszkoreit, J., Jones, L., Gomez, A. N., et al. (2017). Attention is all you need. In *Proceedings of the 31st International Conference on Neural Information Processing Systems* (Red Hook, NY, USA: Curran Associates Inc.), NIPS'17, 6000–6010
